# Gut Microbial Metabolites in Parkinson’s Disease: Implications of Mitochondrial Dysfunction in the Pathogenesis and Treatment

**DOI:** 10.1007/s12035-021-02375-0

**Published:** 2021-04-06

**Authors:** Yixuan Liang, Li Cui, Jiguo Gao, Mingqin Zhu, Ying Zhang, Hong-Liang Zhang

**Affiliations:** 1 Department of Neurology, First Hospital of Jilin University, Changchun, 130021 China; 2 Departments of Laboratory Medicine and Pathology, Neurology and Immunology, Mayo Clinic, Rochester, MN USA; 3 Department of Life Sciences, National Natural Science Foundation of China, Shuangqing Road 83, Beijing, 100085 China

**Keywords:** Parkinson’s disease, Gut microbiota, Microbiota-gut-brain axis, Microbial metabolites, Mitochondrial dysfunction

## Abstract

The search for therapeutic targets for Parkinson’s disease (PD) is hindered by the incomplete understanding of the pathophysiology of the disease. Mitochondrial dysfunction is an area with high potential. The neurobiological signaling connections between the gut microbiome and the central nervous system are incompletely understood. Multiple lines of evidence suggest that the gut microbiota participates in the pathogenesis of PD. Gut microbial dysbiosis may contribute to the loss of dopaminergic neurons through mitochondrial dysfunction. The intervention of gut microbial metabolites via the microbiota-gut-brain axis may serve as a promising therapeutic strategy for PD. In this narrative review, we summarize the potential roles of gut microbial dysbiosis in PD, with emphasis on microbial metabolites and mitochondrial function. We then review the possible ways in which microbial metabolites affect the central nervous system, as well as the impact of microbial metabolites on mitochondrial dysfunction. We finally discuss the possibility of gut microbiota as a therapeutic target for PD.

## Parkinson’s Disease and Mitochondrial Dysfunction

Parkinson’s disease (PD) is a primary neurodegenerative disease and the most common cause of Parkinsonism, which is characterized by the loss of dopaminergic neurons in the substantia nigra pars compacta (SNpc) and the accumulation of α-synuclein into Lewy body inclusions [1]. To date, the etiology and pathophysiology of PD are incompletely understood. Similar to other neurodegenerative disorders, the etiology of PD involves both environmental and genetic factors.

**Fig. 1:**
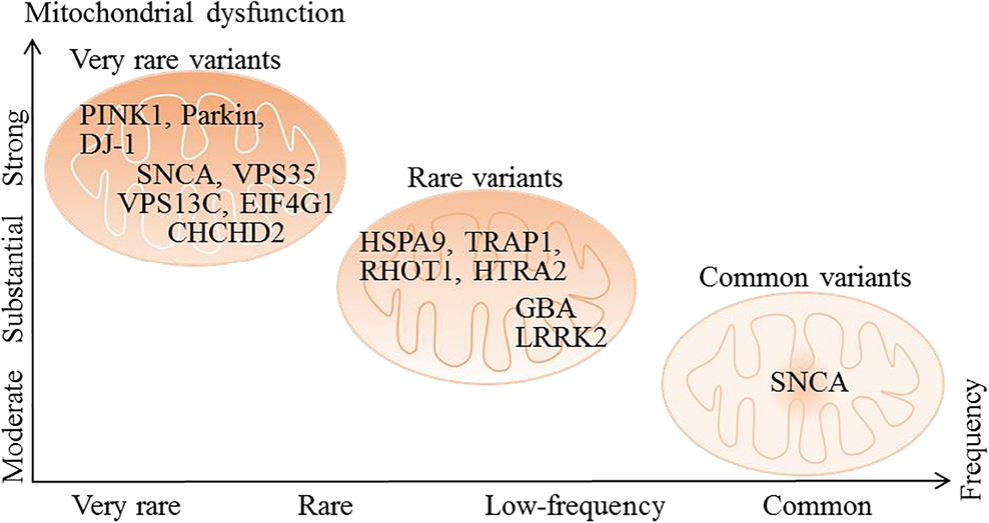
Relationship between PD-related gene mutations and mitochondrial dysfunction. The frequency of PD-related gene mutations is inversely proportional to the severity of mitochondrial dysfunction (modified from [34]). Most variants identified thus far confer relatively small increments in risk, and explain only a small proportion of familial clustering, leading many to question how the remaining, “missing” heritability can be explained [35]

Defects in the mitochondrial respiratory chain complex I have been found in post-mortem brains from patients with sporadic PD [2]. Environmental factors that affect PD etiology such as 1-methyl-4-phenyl-1,2,3,6-tetrahydrodropyridine (MPTP) and rotenone mainly inhibit the function of the mitochondrial respiratory chain by damaging the mitochondrial complex I, leading to bioenergetics failure and subsequent cell death. MPTP and rotenone are being used to induce PD animal and cell models [3]. Familial PD is commonly associated with genetic mutations of proteins including α-synuclein, parkin, phosphatases and tensin homolog deleted on chromosome ten (PTEN)-induced putative kinase 1 (PINK1), DJ-1 (also called Parkinson disease protein 7), and leucine-rich repeat kinase (LRRK)2 [4]. Many of these risk genes for PD are related to mitochondrial function (Table 1). As such, mitochondrial dysfunction may play a central role in the pathogenesis of PD. This aspect has been extensively reviewed elsewhere (see review in [2]). Although familial PD accounts for only a small percentage of PD cases, rare single-gene mutations are highly effective in causing mitochondrial dysfunction, including mutations in PINK1, parkin, and DJ-1. By contrast, more common genetic risk factors such as LRRK2 and some alpha-synuclein (SNCA) genes that encode α-synuclein cause less severe mitochondrial dysfunction for unknown reasons (Fig. 1) [34]. Current research suggests that mitochondrial dysfunction is caused by multiple types of damage, including defects in mitochondrial energy metabolism, disorders of calcium homeostasis, production of reactive oxygen species (ROS), and abnormalities in mitochondrial dynamics. These forms of damage lead to insufficient mitochondrial energy supply, increased expression of mitochondrial-dependent pro-apoptotic pathways, and mitochondrial fragmentation [36].

**Table 1:** Familial PD genes and their potential role in mitochondrial dysfunction (modified from [5])

Genes	Associated mitochondrial dysfunction	References
α-synuclein (Park1/4)	Reduced Complex I activity and oxygen consumption rate Abnormal mitochondrial morphology, Ca^2+^ dyshomeostasis Abnormal ER-mitochondria transport	[6–8]
*Parkin* (Park2)	Reduced mitochondrial respiration, oxidative damage Mitochondrial functional integrity Reduced mitochondrial biogenesis Abnormal mitochondria High mitochondrial ROS	[9–12]
*PINK1* (Park6)	Reduced electron transfer cascade enzyme function Reduced ATP production, Ca^2+^ dyshomeostasis Reduced mitochondrial function, fission Abnormal mitochondria and high mitochondrial ROS Abnormal mitochondrial Ca^2+^ handling	[12–16]
*DJ1* (Park7)	Abnormal mitochondrial morphology Uncoupled mitochondria Glycolytic shift Mutants induce mitochondrial fragmentation	[17–19]
*LRRK2* (Park8)	Reduced ATP production and membrane potential Abnormal mitochondrial fission/fusion Delayed Miro degradation and mitophagy	[20–22]
*ATP13A2* (Park9)	Mutant causes low mitochondrial oxygen consumption rate, reduced ATP synthesis	[23]
*HTRA2* (Park13)	Mitochondrial morphological abnormalities Low respiration, increased sensitivity to apoptosis	[24, 25]
*FBXO7* (Park15)	Impaired ubiquitin-proteasome system, reduced mitophagy leading to accumulation of dysfunctional mitochondria Mitochondrial accumulation of aggregates	[26, 27]
*VPS35* Park17	Mitochondrial fragmentation, reduced oxygen consumption	[28]
*CHCHD2* (Park22)	Decreased complex I activity, respiration, increased ROS transcription factor for complex IV subunit cytochrome c oxidase 4I2 Dysregulated apoptosis	[29–31]
*PLA2G6*	Decreased mitochondrial membrane potential and function	[32]
*GBA*	Reduced macro-autophagy leading to accumulation of dysfunctional mitochondria	[33]

*PD*, Parkinson’s disease; *ER*, endoplasmic reticulum; *ROS*, reactive oxygen species; *ATP*, adenosine triphosphate

In this narrative review, we first summarize the associations among gut microbiota, mitochondrial dysfunction, and PD pathogenesis. We then review possible ways in which microbial metabolites affect the central nervous system (CNS) and mitochondrial function. Finally, we discuss the potentials of gut microbiota as a therapeutic target for PD.

## Association of PD Pathogenesis with Gastrointestinal Dysfunction and Gut Dysbiosis

The understanding of gastrointestinal (GI) dysfunction in patients with PD is increasing. The enteric nervous system (ENS) and vagus nerve may be affected as early as the prodromal phase of the disease [37]. Clinically, constipation, a common non-motor symptom of PD, can appear several years earlier than motor symptoms do. Pathologically, the hallmark protein of PD, i.e., α-synuclein, accumulates first in the intestinal submucosal plexus [38]. Patients with PD have increased expression of proinflammatory cytokines and glial markers in colonic biopsies, which may intensify the accumulation of α-synuclein [39]. Hypothetically, abnormal α-synuclein begins to accumulate in the GI tract is transported to the CNS via the reverse axon transport system in the vagus nerve [40]. Except for direct damage to dopaminergic neurons, α-synuclein deposited in the brain promotes a neuroinflammatory response furthering aggravating neurodegeneration [41].

The gut microbiota is composed of a variety of microorganisms including bacteria, viruses, and eukaryotes [42]. As an extension of the concept of the gut-brain axis, the microbiota-gut-brain axis represents a complex multidirectional cross-talk system between the gut microbiota, the ENS, and the CNS, which integrates immunological, neuroendocrine, and neurological processes [43]. Although the composition of the gut microbiota is relatively stable in adulthood, it can still be disrupted by factors such as diet, infection, lifestyle, and the environment [44], as illustrated by the term dysbiosis. The association between gut dysbiosis and changes in microbial metabolites in PD was extensively reviewed elsewhere [45]. Importantly, the regulation of motor deficits and neuroinflammation by the gut microbiota has been confirmed in a murine model of PD [46].

Changes in the gut microbiota of PD patients may increase the risk of weakened intestinal mucosal protection and bacterial translocation. For example, *Akkermansia muciniphila* in PD patients is more abundant at the genus level. The increase in *Akkermansia muciniphila*, which degrades mucin, may be one of the reasons for the increased intestinal permeability in patients with PD [47]. Nevertheless, the increase in *Akkermansia* is not necessarily unfavorable. Studies have shown that niacinamide (NAM) produced by *Akkermansia* has potential therapeutic significance in neurodegenerative diseases [48]. These conflicting findings regarding the role of *Akkermansia* require further investigation.

Short-chain fatty acids (SCFAs) provide energy to colon cells and prevent the increase of intestinal mucosal permeability to reduce bacterial translocation [49]. The abundance of gut microbiota that produce SCFAs at the genus or species level is lower in patients with PD, and a significant decrease in SCFAs has been observed in the intestines of these patients. Conversely, SCFAs promote α-synuclein accumulation and aggravate PD motor symptoms at a certain dose in murine models [46].

## Microbial Metabolites and Mitochondrial Dysfunction in PD: Is There a Link?

Multiple lines of evidence support the remote regulation of CNS function by gut microbiota. For example, germ-free (GF) mice showed less anxiety than specific pathogen-free (SPF) mice, along with decreased expression of receptors related to anxiety and learning in the amygdala [50]. Additionally, antibiotic treatment for gut microbiota effectively improved the symptoms of patients with hepatic encephalopathy [51]. Using mice that overexpress α-synuclein, researchers found that gut microbiota were required for α-synuclein pathology and microglia activation and also played a role in the development of motor deficits [46]. Antibiotic treatment ameliorated, while microbial recolonization promoted, the pathophysiology in adult animals [46]. Importantly, oral administration of specific microbial metabolites to GF mice promoted neuroinflammation and motor symptoms [46].

**Fig. 2:**
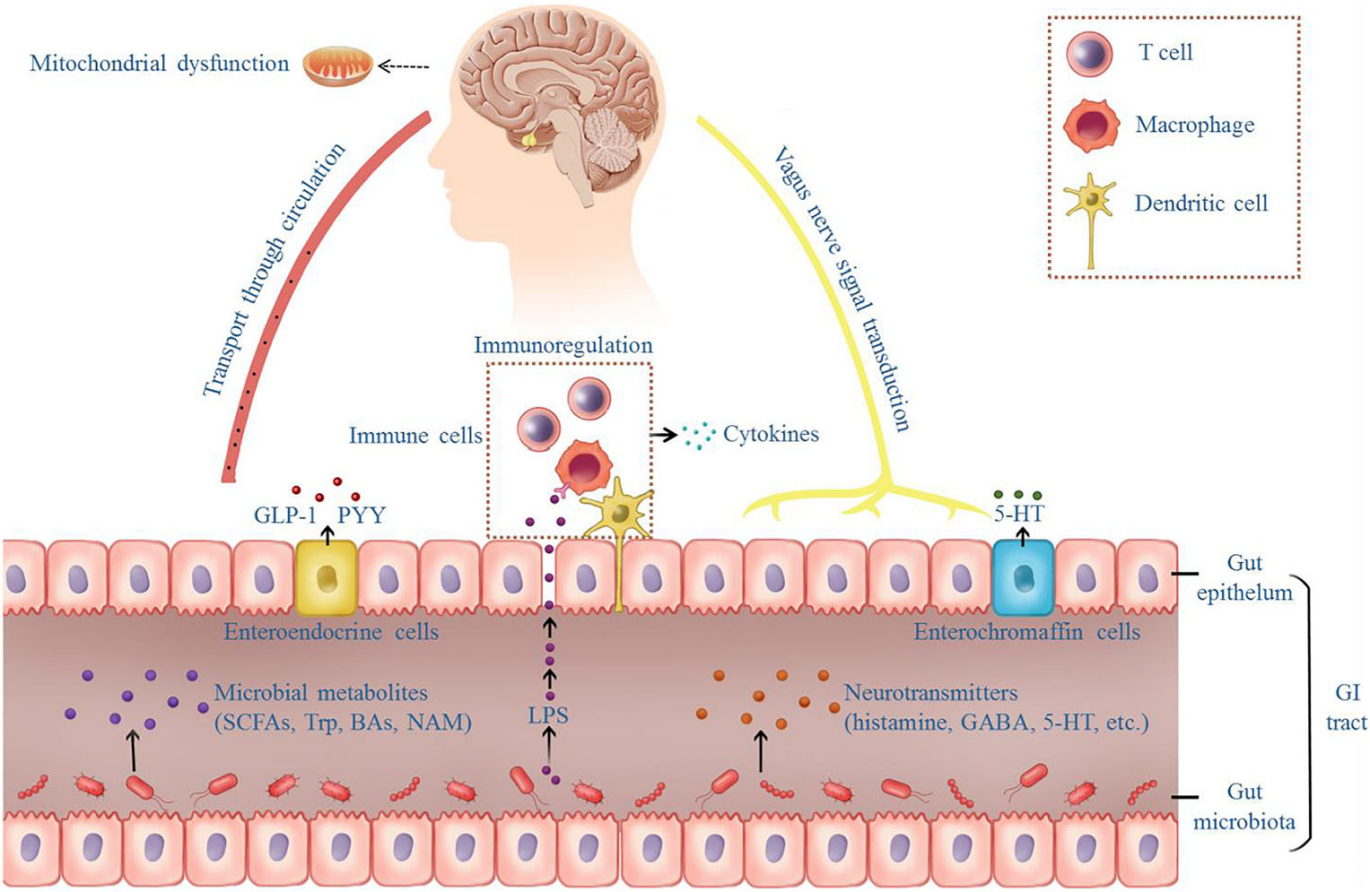
Two main bidirectional information communication pathways between the gut and the brain. Under pathophysiological conditions, gut dysbiosis may alter intestinal permeability, increase bacterial translocation, and initiate TLR-mediated intestinal inflammation. Proinflammatory factors involved in local intestinal inflammatory reactions and disordered gut inflammation may reach the brain to induce mitochondrial dysfunction. The gut-brain communication via microbial metabolites mainly implicate two anatomical pathways: (1) The gut microbial metabolites reach the submucosa of the intestine; enter the enterohepatic circulation, pulmonary circulation, and systemic circulation in turn; and finally reach the brain, and (2) intestinal signals signalled by the intestinal submucosal nerve plexus propagate along the vagus nerve to the CNS. 5-HT, 5-hydroxytryptamine; BAs, bile acids; CNS, central nervous system; GABA, γ-aminobutyric acid; GLP-1, glucagon-like peptide 1; NAM, niacinamide; PYY, peptide YY; SCFAs, short-chain fatty acid; TLR, toll-like receptor; Trp, tryptophan

The communication between the gut microbiota and CNS via microbial metabolites mainly occurs in two ways (Fig. 2): (1) The gut microbial metabolites reach the submucosa of the intestine; enter the enterohepatic circulation, pulmonary circulation, and systemic circulation in turn; and finally reach the brain [52], and (2) intestinal signals stimulate the intestinal submucosal nerve plexus and propagate along the vagus nerve to the CNS. Animal studies further confirmed the existence of an intestinal ascending pathway, including the right nodose, the parabrachio-nigral pathway, and its targets in the dorsal striatum, which connects intestinal signals with dopamine (DA) release in the substantia nigra [53].

The gut microbiota converts substrates into different metabolites including SCFAs, NAM, bile acids (BAs), and neurotransmitters [54–57]. As a consequence of the disruption of the gut microbiota, normal signal transmission from the gut microbiota to the brain is disrupted, which may contribute to the pathological mechanism of neuropsychiatric diseases [45]. Some researchers suggest that the imbalance of gut microbiota, which leads to an increase in damaging factors, results in neurodegenerative processes, with mitochondria as the target [58]. In this section, we review several microbial metabolites related to PD and focus on their impact on mitochondrial dysfunction (Table 2).

**Table 2:** Microbial metabolites related to the pathogenesis of PD

Microbial metabolites		Bacterial genera	Gut-brain communication	Mechanisms in the pathogenesis of PD
NAM		63% human gut bacteria genomes [56]	Circulation [59]	NAM acts as a precursor of NAD to provide coenzymes required by the mitochondrial respiratory chain and protect mitochondria from damage [60].
BAs		*Clostridium*, *Eubacteria* [61]	Circulation [62]	TUDCA and UDCA promote mitophagy to protect mitochondrial function [63, 64].
SCFAs		Most gut anaerobes: acetate *Firmicutes*: butyrate *Bacteroidetes*: propionate [42, 65]	Circulation and vagus nerve [66]	SCFAs act as energy substrates for mitochondria and promote mitochondrial fusion [67, 68]
Tryptophan		*Escherichia coli* Tryptophan is primarily dependent on exogenous uptake [69]	Circulation [70]	1. KP: The neuroprotective metabolites of KP such as KYNA, picolinic acid, and NAD^+^, and neurotoxic products such as QA, 3-HK [71]. 2. Serotonin pathway: 5-HT in the brain is related to memory, mood, cognitive function, and severity of resting tremor in PD [72, 73]. Melatonin provides a substrate for the mitochondrial respiratory chain and reduces oxidative stress and apoptosis [74, 75].
Neurotransmitters	Histamine	*Escherichia coli* *Morganella morganii* *Lactobacillus Lactococcus* *Streptococcus* *Pediococcus* *Enterococcus* spp [76, 77]	Vagus nerve [78]	Histamine activates H2R to increase mitochondria-dependent apoptosis [79].
	GABA	*Lactobacillus* *Bifidobacterium* [80]	Vagus nerve [81]	GABA can accurately control the quantity of Ca^2+^ that enters the cell to protect mitochondria from damage caused by Ca^2+^ overload [82].

*PD*, Parkinson’s disease; *NAM*, niacinamide; *NAD*, nicotinamide adenine dinucleotide; *BAs*, bile acids; *TUDCA*, tauro ursodesoxy cholic acid; *UDCA*, ursodesoxy cholic acid; *SCFA*, short-chain fatty acid; *KP*, kynurenine pathway; *QA*, quinolinic acid; *3-HK*, 3-hydroxykynurenine; *KYNA*, kynurenic acid; *5-HT*, 5-hydroxytryptamine; *H2R*, histamine 2 receptor; *GABA*, γ-aminobutyric acid

### SCFAs

SCFAs are exclusively produced by the gut microbiota through saccharolytic fermentation. Acetate is produced by most gut anaerobes, propionate is mainly produced by *Bacteroidetes*, and butyrate is mainly produced by *Firmicutes* [42, 65]. In the gut, SCFAs are required for peristalsis and to maintain the intestinal barrier [83]. Stool samples from PD patients showed reduced levels of SCFA-producing bacteria though the significance of the pathogenic mechanism remains unknown [84]. In a rotenone-induced drosophila model of PD, SCFAs had a dose-dependent neuroprotective effect on improving motor symptoms by upregulating histone acetylation to increase DA levels [85]. SCFAs can also upregulate the expression of tyrosine hydroxylase (TH) mRNA by inhibiting histone deacetylase (HDAC) [86]. HDAC is considered to optimize mitochondrial function by reducing oxidative stress and enhancing mitochondrial oxidative phosphorylation [87]. In addition, the colon cells from GF mice show energy deficiency; however, the addition of butyrate can prevent mitochondrial energy metabolism defects by providing acetyl coenzyme A (acetyl-CoA), which participates in the mitochondrial tricarboxylic acid cycle [67]. Oral butyrate alleviated the damage of mitochondria in the brains of d-amphetamine-treated rats [88], suggesting that SCFAs in the intestine can be systematically circulated to the brain to improve mitochondrial function in the CNS.

**Fig. 3:**
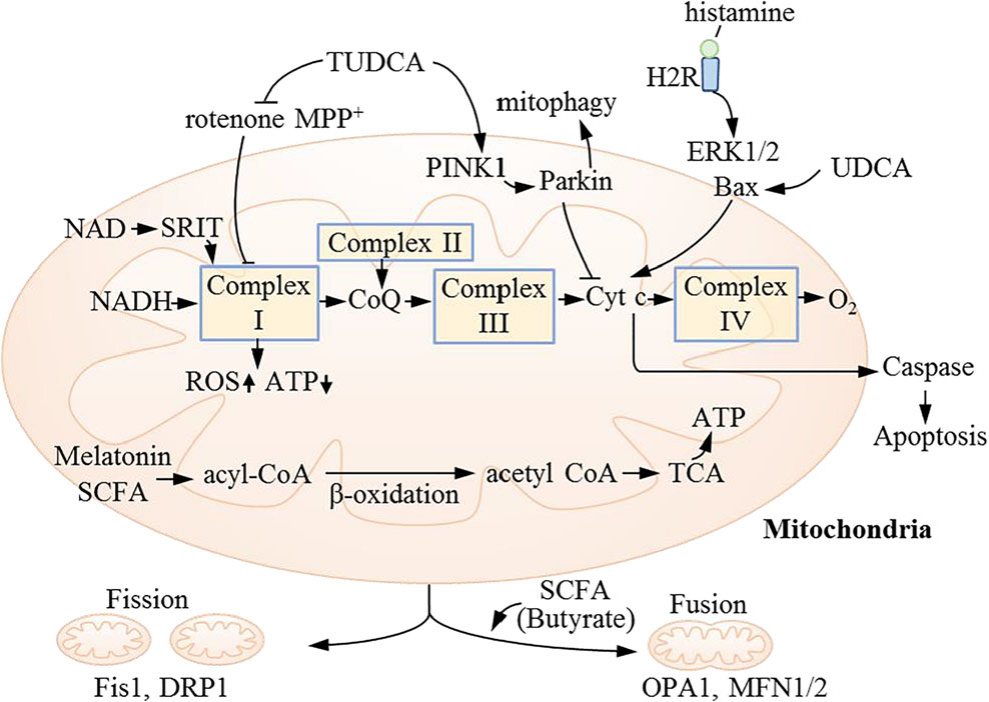
Microbial metabolites affect neuronal mitochondrial function through different pathways in PD. Mitochondria are responsible for the production of adenosine triphosphate (ATP) via the combined efforts of the tricarboxylic acid cycle and the respiratory chain/oxidative phosphorylation system (OxPhos). The respiratory chain is a set of biochemically linked complexes, namely complexes I, II, III, and IV with two electron carriers, namely ubiquinone/CoQ and Cyt *c*. The energy stored in food was used by the respiratory chain to generate a proton gradient across the mitochondrial inner membrane, while at the same time transferring electrons to oxygen and producing water. The energy of the proton gradient drives ATP synthesis via ATP synthase (complex V). Gut microbial metabolites have multiple regulatory effects on the mitochondrial function, including regulating complex I (TUDCA, NAM, NAD), the Cyt-*c*-induced caspase-dependent apoptosis pathway (histamine), beta-oxidation and acetylation (SCFA), PINK1/Parkin-induced mitophagy (TUDCA), and mitochondrial dynamics (SCFA). MPTP and rotenone mainly inhibit the function of the mitochondrial respiratory chain by damaging mitochondrial complex I, leading to bioenergetics failure and subsequent cell death, and they are often used as inducers of PD animal and cell models. TUDCA, a taurine-bound form of UDCA, is an anti-apoptotic agent by up-regulating mitophagy. TUDCA can upregulate the expression of PINK1 and parkin to accelerate the clearance of damaged mitochondria, promoting the survival of damaged neurons. Butyrate can prevent mitochondrial energy metabolism defects by providing acetyl-CoA, which participates in the mitochondrial tricarboxylic acid cycle. Mitochondria are dynamic organelles that constantly undergo fission and fusion. The transition to fusion optimizes the function of mitochondria and helps maintain long-term bioenergy capabilities. Conversely, the transition to fission will result in the production of large amounts of mitochondrial fragments and autophagy of the damaged mitochondria. Butyrate increases the expression of fusion protein mRNA and the transcription of fission protein continues to decrease. The guiding effect of SCFAs on mitochondrial dynamics improves the bioenergetic efficiency. acyl-CoA, acyl-coenzyme A; acetyl-CoA, acetyl coenzyme A; ATP, adenosine triphosphate; CoQ, coenzyme Q; Cyt *c*, cytochrome *c*; DRP1, dynamin-related protein 1; ERK1/2, extracellular signal-regulated kinase 1/2; Fis1, fission 1; H2R, histamine 2 receptor; *MPP^+^, 1-methyl-4-phenyl pyridinium ion; MPTP, 1-methyl-4-phenyl-1,2,3,6-tetrahydrodropyridine; NAD, nicotinamide adenine dinucleotide; NADH, reduced form of nicotinamide-adenine dinucleotid; OPA1, optic atrophy 1; PD, Parkinson’s disease; PINK1, phosphatases and tensin homolog deleted on chromosome ten-induced putative kinase 1; ROS, reactive oxygen species; SCFA, short-chain fatty acid; SIRT, sirtuin; TCA, tricarboxylic acid cycle; TUDCA, tauro ursodesoxy cholic acid; UDCA, ursodesoxy cholic acid. *MPP+ is an active metabolite of MPTP, a neurotoxin capable of causing selective destruction of dopaminergic neurons

Mitochondria are dynamic organelles that constantly undergo fission and fusion. The transition to fusion optimizes the function of the mitochondria and helps maintain their long-term bioenergy capabilities. Conversely, the transition to fission results in the production of large amounts of mitochondrial fragments and autophagy of the damaged mitochondria [68]. Butyrate treatment significantly increased the expression of fusion protein mRNA, and the transcription of fission protein continued to decrease in liver specimens from mice [68]. The guiding effect of SCFAs on mitochondrial dynamics improves the bioenergetic efficiency (Fig. 3).

### Neurotransmitters

Neurotransmitters play a significant role in GI physiology. DA, γ-aminobutyric acid (GABA), and serotonin (5-hydroxytryptamine, 5-HT) influence gut motility, nutrient absorption, the innate immune system in the GI tract, and the microbiome [89]. Neurotransmitter levels may be altered by GI disturbances in patients with PD. According to the literature, enteric neurotransmitters that affect CNS signal transmission are more likely to be induced through vagal signaling. Of note is that the blood-brain barrier (BBB) is damaged in PD patients [90]; therefore, gut-derived neurotransmitters may enter the circulation and cross the damaged BBB to the brain [91, 92].

Histamine-producing bacteria isolated from stool samples include *Escherichia coli*, *Morganella morganii*, and *Lactobacillus vaginalis* [93]. *Lactobacillus*, *Lactococcus*, *Streptococcus*, *Pediococcus*, and *Enterococcus* spp. all have histidine decarboxylase activity and can produce histamine [76]. In the stool of PD patients, histamine-producing *Lactobacillus*, *Streptococcus*, and *Enterococcus* are more abundant compared to that in the control group [47, 94]. Histamine concentrations were significantly increased in the putamen, SNpc, and globus pallidus in PD patients [95]. As these regions play a crucial role in motor function, the selective increase of histamine is suggestive of the pathological changes in PD [95]. Histamine binds to four types of receptors and mediates a wide range of physiological effects throughout the body. The receptors widely expressed in the CNS determine the universal function of histamine. Histamine 1 receptor (H1R) is the main histamine receptor in the brain and has an excitatory effect [96]. H2R activation results in the excitation of neural cells [96]. H3R is located on the axons of neurons and dendrites, providing negative feedback to inhibit histamine synthesis and the release of histamine and other neurotransmitters [97]. H4R is present in microglia, but its function is unclear [97]. Due to the extensive distribution of histamine receptors and the diversity of their functions, the mechanism of histamine receptors in PD remains elusive. H2R activation promotes cell apoptosis through mitochondria-dependent apoptotic pathways [79]. H2R activation increases the expression of the pro-apoptotic protein Bax in cardiomyocytes. The translocation of Bax protein to the outer mitochondrial membrane increases the permeability of the mitochondrial membrane, consequently promoting the release of pro-apoptotic factors such as cytochrome *c*. In addition, the expression of extracellular-regulated protein kinases 1/2 (ERK1/2) is also increased by activated H2R to promote the release of cytochrome *c* [79, 98]. Cytochrome *c* is released into the cytoplasm, leading to the expression of caspase-3, which induces the apoptosis of cells [79]. Elevated histamine aggravates cell apoptosis by binding to H2R. In the PD mouse model, the H2R antagonist ranitidine reduced the expression of ERK1/2 in the striatum [99]. These results suggest that blocking H2R may be a beneficial treatment option for PD by reducing the expression of mitochondrial-dependent apoptotic pathways (Fig. 3).

Bacteroides, Parabacteroides, and Escherichia species actively express the GABA production pathway in healthy human stool and produce GABA in the intestine [100]. The abundance of Parabacteroides and Bacteroides was found to be elevated in PD patients compared to that in the control group [101, 102]. Magnetic resonance spectroscopy revealed that the increase of GABA in the pons, basal ganglia, and thalamus is associated with the degree of bradykinesia and rigidity in PD patients [103]. Treatment with *Lactobacillus rhamnosus* resulted in the increase of GABA levels in the brain of mice, yet the neurochemical effects were not observed in vagotomized mice [104, 105], which demonstrates the vagus nerve acts as a significant pathway in the remote regulation of the brain by intestinal microbes. Indeed, individuals that underwent full truncal vagotomy had a lower risk for subsequent PD, strongly implicating the vagus nerve in the pathogenesis of PD and again corroborating the involvement of an enteric pathogen or toxin.

A low calcium buffering capacity results in the loss of dopaminergic neurons in the SNpc in patients with PD. GABA can control the quantity of Ca^2+^ that enters a cell, which may stabilize neuronal activity at the cellular and systemic levels [82]. A mitochondrial matrix can be used as a temporary buffer pool for intracellular Ca^2+^. The removal of Ca^2+^ from mitochondria and cytoplasm requires a large amount of cellular energy [106]. In experiments with cardiomyocytes, a high intracellular Ca^2+^ load induced the opening of mitochondrial permeability transition pores, resulting in the release of pro-apoptotic factors into the cytoplasm, increased oxidative stress, and abnormal mitochondrial membrane potential, ultimately leading to cell apoptosis [107]. Therefore, GABA may protect mitochondrial function, reduce oxidative stress, and consequently prevent the death of dopaminergic neurons by preventing Ca^2+^ from entering dopaminergic neurons.

### Tryptophan and 5-HT

Tryptophan is primarily dependent on exogenous uptake, and a small portion is produced by gut microbiota such as *Escherichia coli*. Tryptophan can be metabolized by the gut microbiota to aromatic hydrocarbon receptor ligands as well as through the kynurenine pathway (KP) or the 5-HT pathway [69]. Tryptophan metabolized via the KP can produce neuroprotective metabolites, including kynurenic acid (KYNA), picolinic acid, and NAD, and neurotoxic metabolites, including quinolinic acid (QA) and 3-hydroxykynurenine (3-HK) [71]. Tryptophan is more likely to be metabolized into neurotoxic compounds in PD patients. The level of KYNA is decreased in the putamen, SNpc, and the frontal cortex [108], while QA in plasma and 3-HK in the SNpc and putamen are increased in PD patients [109]. Tryptophan metabolized to 3-HK and QA may be secondary to the inhibitory effects of mitochondrial complex I, and the accumulation of 3-HK and QA can aggravate neurotoxicity and oxidative stress. This vicious circle further aggravates mitochondrial damage, subsequently leading to the loss of SNpc dopaminergic neurons [109].

In addition, 5-HT synthesis in the CNS can be regulated by tryptophan [110], which affects behavior, emotion, and memory [111]. In PD, the severity of resting tremor and decreased cognitive function is associated with the degeneration of 5-HT neurons [72, 112]. Interestingly, as a metabolite of the 5-HT pathway, melatonin has been shown to relieve the non-motor symptoms of PD [113]. Mechanistically, melatonin reduces the death of dopaminergic neurons by decreasing oxidative stress and the expression of mitochondrial-dependent apoptotic pathways [74]. Melatonin also provides acetyl-CoA to mitochondria, which enhances the function of mitochondrial bioenergetics [75]. Therefore, melatonin plays a protective role in PD by optimizing mitochondrial function. Regulation of the exogenous intake and metabolic pathway of tryptophan may be a potential treatment target in PD.

### BAs

BAs, including chenodeoxycholic acid (CDCA) and cholic acid, are synthesized in the liver, stored in the gallbladder, and released into the duodenum under the stimulation of food [57]. Most BAs are reabsorbed in the intestine, but a small proportion enter the colon and are biotransformed into secondary BAs [57]. Ursodeoxycholic acid (UDCA), the 7 beta-hydroxy epimer of CDCA, is present in trace amounts [57]. An estimated millesimal bacterium in the colon, which belongs to the genus *Clostridium*, is able to transform primary BAs to secondary BAs [61]. In the plasma of PD patients, the glycine-bound form of UDCA was found to be lower [114], whereas liver-derived primary bile acid, bacterially generated secondary bile acid, and conjugated bile acids were elevated. Notably, both L-dopa and combinational treatments could alleviate the elevations of BAs in PD patients [115].

Most types of primary and secondary BAs are found in the human brain [62]. UDCA or tauro ursodesoxy cholic acid (TUDCA) treatment improved motor performance, ameliorated mitochondrial dysfunction and neuroinflammation, and prevented the decline of striatal dopamine content in various PD models. TUDCA, a taurine-bound form of UDCA, is an anti-apoptotic agent that upregulates mitophagy [63]. TUDCA can upregulate the expression of PINK1 and parkin in SH-SY5Y cells to accelerate the clearance of damaged mitochondria, promoting the survival of damaged neurons [63]. Moreover, in the PD model of rats, UDCA treatment rescued the DA content in the striatum and relieved the motor symptoms by downregulating the expression of Bax and maintaining the integrity of the mitochondrial membrane. This effect was accompanied by a decrease in the expression of the pro-apoptotic pathway including caspase-9, caspase-3, and caspase-8 [64]. We postulate that TUDCA and UDCA maintain mitochondrial function to reduce the damage of dopaminergic neurons by accelerating the clearance of damaged mitochondria and reducing the expression of pro-apoptotic pathways. UDCA and TUDCA appear to have the potential to manage diseases associated with elevated apoptosis, including neurodegeneration [116] (Fig. 3).

### NAM

NAM, the amide form of vitamin B3 (niacin), is a precursor of nicotinamide adenine dinucleotide (NAD) [117]. Systematic evaluation of the genomes of 256 common human gut bacteria revealed that niacin biosynthesis capability is present in 63% of human gut microbiota genomes, including those of *Bacteroidetes*, *Clostridium*, *Proteobacteria*, *Actinobacteria*, and *Firmicutes* [56]. The level of NAD in the plasma of PD patients is reduced, but the relationship with PD is still unclear [118]. In the drosophila model of PD, NAM supplementation significantly increased the level of NAD to enhance motor function, to relieve oxidative stress, and to maintain mitochondrial function [60]. NAD is an essential coenzyme in the mitochondrial respiratory chain and serves as a substrate for various enzymes. NAD-consuming enzymes, such as the deacetylase sirtuin (SIRT) family and poly (ADP-ribose) polymerases (PARPs), depend on NAD to exert their biological effects [119].

In the dopaminergic neuron model of PD, NAD levels and SIRT activity were significantly reduced. SIRT has been proven to exert anti-aging and antioxidant effects. In macrophages, SIRT regulates mitochondrial function through the deacetylation of complex I and plays a key role in enhancing antioxidant activity and resisting the increase in mitochondrial ROS [120]. Since it depends on NAD to function, the protective effect of SIRT is significantly weaker in dopaminergic neurons [121].

PARPs are enzymes involved in the nuclear DNA repair of healthy cells. Excessive activation of PARPs is related to the destruction of mitochondrial structure and the toxicity of dopaminergic neurons [122]. In the PINK1 mutant drosophila model, PARPs were found to be overexpressed. The mitochondrial damage caused by PARPs was rescued by adding NAM to the diet [122]. The above evidence demonstrates the therapeutic potential of NAM in PD models related to mitochondrial dysfunction (Fig. 2).

### LPS

The intestinal barrier is maintained by a series of tight junction proteins, including zonula occluden (ZO)-1 [123]. ZO-1 expression in patients with PD is significantly lower than that in healthy controls. Lipopolysaccharide (LPS) is an endotoxin produced by Gram-negative bacteria. Under physiological conditions, the integrity of the intestinal barrier prevents bacteria and LPS from contact with the epithelial cells. An impaired intestinal barrier allows bacteria and LPS to penetrate the epithelium into the circulation under the intestinal mucosa [123]. The level of LPS markers in the colon and plasma of PD patients is significantly increased, which results in the activation of a series of inflammatory reactions, manifesting as an increase in the T cell transport to the colonic mucosa and the number of toll-like receptor 4 (TLR4)-positive cells (such as dendritic cells and macrophages), which have been shown to mediate inflammation [123].

LPS can be used to induce the animal model of PD. Furthermore, rotenone treatment in TLR4-knockout (KO) mice resulted in less intestinal inflammation, intestinal and motor dysfunction, neuroinflammation, and neurodegeneration [123], which highlight the involvement of inflammation in the pathogenesis of PD and communication between the intestine and brain. The BBB prevents neurotoxic plasma components, blood cells, and pathogens from entering the brain. α-synuclein crosses the BBB bidirectionally, which could signify an important contributory event in PD pathogenesis [124]. α-synuclein influx is increased following LPS-induced BBB breakdown [124], suggesting that the high levels of α-synuclein produced peripherally can enter the brain in the presence of BBB breakdown, which may also contribute to the development of PD pathology [125].

As a classic proinflammatory substance, LPS also seriously affects mitochondrial function. LPS significantly induced mitochondrial ROS production in the microglia model, and ROS further activated the inflammation of the microglia [126]. LPS seems to induce mitochondrial dynamics to be more prone to fission and mitochondrial fragmentation [127]. In macrophages, LPS treatment downregulates PINK1-induced mitochondrial autophagy, leading to the accumulation of dysfunctional mitochondria [128]. Taken together, LPS may contribute to pathological changes in PD, at least partially, by inducing mitochondrial damage and aggravating apoptosis in dopaminergic neurons.

## Can Gut Microbiota Serve as a Therapeutic Target for PD?

Gut microbiota are affected by various endogenous and exogenous factors. The recovery and maintenance of gut microbiota may represent a therapeutic option for diseases related to dysbiosis. Targeting the gut microbiota using probiotics, antibiotics, and fecal microbial flora transplantation may restore the composition of the gut microbiota, replenish beneficial metabolites, and reduce harmful metabolites to address the pathophysiology and mitigate the symptoms of PD.

### Probiotics

Probiotics are living microbial preparations that are beneficial to human health [129]. In mice, administration of *Lactobacillus rhamnosus* increased the expression of GABA in the brain and reduced anxiety and depression-related behaviors [104, 105]. A mixed probiotics preparation (*Bifidobacterium lactis*, *Lactobacillus acidophilus*, *Lactobacillus paracasei*, and *Lactobacillus plantarum*) raised the level of SCFAs in in vitro experiments on the human colon [130]. In patients with mental illness, some psychobiotics defined as live bacteria are considered to relieve mental symptoms by promoting the synthesis of endogenous neurotransmitters, such as GABA, catecholamines, and 5-HT [131]. Several studies have revealed the benefits of probiotics in patients with PD, including the alleviation of constipation and motor symptoms [132]. In an in vivo nonhuman primate intestinal loop model of acquired immune deficiency syndrome, the provision of probiotics helped repair the intestinal barrier to reduce bacterial translocation through the restoration of mitochondrial function and an increase in the level of SCFAs [133]. Taken together, controlling gut microbiota via probiotics may provide SCFAs to the host to exert a protective effect in PD.

### Prebiotics

Prebiotics are soluble dietary fibers that stimulate the growth of gut commensal microbiota to combat disease and maintain health. In mice, long-term use of fructo-oligosaccharides (FOS) and galactooligosaccharides (GOS) significantly improved anxiety and depression-related behaviors by increasing SCFA-producing bacteria [134]. Combined use of prebiotics and probiotics alleviated mitochondrial dysfunction in the brain of mice fed on a high-fat diet [135], indicating that increasing beneficial bacteria in the intestine has the potential to attenuate CNS disease. Consumption of fermented milk containing prebiotic fiber improved constipation in PD patients [136]. Prebiotics may improve the pathology of PD by stimulating the colonization of beneficial microorganisms in the intestine and promoting the secretion of SCFAs.

### Antibiotics

Antibiotics can kill or inhibit microorganisms at low concentrations. Antibiotics can also inhibit the accumulation of abnormal proteins and improve mitochondrial function, which might be beneficial for the treatment of neurodegenerative disease [137]. Many antibiotics such as rifampicin and ceftriaxone may be beneficial in the treatment of PD [138, 139]. Eradication of *Helicobacter pylori* improves levodopa absorption and PD motor symptoms [140]. However, the resistance of microorganisms to antibiotics is an important issue in clinical applications. Furthermore, exposure to certain oral antibiotics may increase the risk of PD possibly due to the long-term effects of antibiotics on the composition of the human gut microbiota [141]. Therefore, the development of antibiotic derivatives that have neuroprotective effects without antibacterial activity is critical for their application in the treatment of neurodegenerative disease.

### FMT

Fecal microbial flora transplantation (FMT) represents a therapeutic strategy by which the feces of healthy donors are delivered to patients to achieve a therapeutic effect by restoring a stable gut microbial environment [142]. FMT is a more efficient approach than probiotic interventions to rebuild a healthy gut microbiota structure. In MPTP-induced mice model of PD, FMT alleviated intestinal microbial disorders and increased striatal DA and 5-HT content [143]. FMT is used to treat GI diseases such as inflammatory bowel disease and irritable bowel syndrome [144]. Patients with non-GI diseases such as multiple sclerosis, myoclonus-dystonia, autism, and depression may also benefit from FMT [145]. Numerous clinical trials on FMT treatment for PD are being conducted.

### Single Microbial Metabolite Supplement Therapy

To date, there is no reliable clinical data to prove the possible influence of probiotic treatment on motor symptoms or PD progression. Although FMT is an attractive technique, many questions regarding its safety and effectiveness remain to be answered before it can be applied in PD treatment. As mentioned above, there are some microbial metabolites that can exert neuroprotective effects in PD by improving mitochondrial function, suggesting that targeting and regulating microbial metabolites may be a potential therapeutic direction.

ClinicalTrials.gov is a database of privately and publicly funded clinical studies conducted around the world. In an ongoing experiment (No. NCT02967250) registered at ClinicalTrials.gov, researchers will evaluate the cortical bioenergy spectrum and ATPase activity of PD patients after continuous oral administration of UDCA using magnetic resonance spectroscopy. The researchers hypothesized that repeated oral administration of UDCA would increase brain ATP levels in PD patients. The theoretical basis for this experiment is the protective effect of UDCA on mitochondria, as identified in cell and animal experiments. Another ongoing clinical trial aims to assess the effects of oral NAM (No. NCT03568968) on the symptoms and neurometabolic profile of PD patients. The trial builds upon experimental data from PD cells and animal models indicating that NAM supplementation maintains mitochondrial function by increasing NAD levels to enhance the metabolism of dopaminergic neurons, thereby improving the PD pathology. The results of these experiments are anticipated, and more experiments are needed to verify the therapeutic effects of UDCA and NAM in PD patients in the future.

## Conclusions and Future Perspectives

Both genetic and environmental factors may cause mitochondrial dysfunction in the pathogenesis of PD. A large number of studies have demonstrated that the intervention of microbial metabolites changes physiological activities in the brain, including neuroinflammation and mitochondrial function, which involves microbial metabolites, abnormal protein aggregation, and microglial activation. Microbial metabolites influence mitochondrial function, which appears to be a key mechanism responsible for the progressive loss of dopaminergic neurons. Although a causal relationship remains to be uncovered, clinical and pathological evidence indicates that the intestinal microenvironment plays a role in the early stage of PD.

Cost-effective and rapid sequencing as well as other research techniques have facilitated the characterization of the gut microbiota in PD. However, a number of challenges remain. First, a clear definition of a healthy microbiome is required to differentiate microbiomes that lead to pathology and provide therapeutic targets for PD. As the mammalian microbiome is complex and composed of 300 to 1000 bacterial species with a total number that exceeds that of host cells, isolating any bacterial components for causative or mechanistic analyses would be extremely difficult. Second, identification of relevant metabolites using techniques such as metabolomics may indicate how alterations in the gut microbiota, environment, diet, and drugs influence metabolite levels. Despite the substantial progress which has been made in growing diverse microorganisms of the microbiota, 23–65% of species residing in the human gut remain uncultured, which is an obstacle to understanding their biological roles [100]. A likely reason for this failure is the absence of key growth factors in artificial media that can be provided by neighboring bacteria in situ. Third, research on the gut microbiota and PD may be confounded by factors such as tremendous individual compositional variations, diet, and drugs. The composition and functionality of the gut microbiota are altered in patients with PD. There is an unmet need for large longitudinal studies combining in-depth phylogenetic analysis with a comprehensive phenotypic characterization of patients with PD using ’omics (meta-genomics, metabolomics, transcriptomics, and meta-transcriptomics). Fourth, translation of basic research into clinically relevant effects in humans must be a priority. However, applying the results of basic research to humans has limitations. For example, the fixation of mammalian tissues for microscopic observations undoubtedly interferes with the regulation of mitochondrial dynamics and may yield limited or misleading results. These limitations may also be related to the host-specific interactions with microbiota. Finally, the current research does not yet include the pathophysiological effects of microbiota other than the gut, oral cavity, and nasal cavity on neurodegenerative diseases. Further research should explore the nervous system role of microbiota in other parts of the human body such as the skin.

Future therapeutic interventions will likely be individualized to accommodate the variety in gut microbiota configuration and composition among human populations. Probiotics seem to have limited effects in regulating the gut microbiota, and the FMT research field is relatively lacking. FMT therapy may be able to better improve the symptoms of PD and delay the progression of the disease. However, FMT treatment still faces the problems of individualized differences, uncertain duration of effect, and a possible need for repeated transplantation. Besides the aforementioned methods to restore gut microbiota composition, the regulation of metabolites (either supplementation of good metabolites or reduction of bad metabolites) seems more controllable and targeted. Ongoing clinical trials are being conducted to explore whether regulating metabolites such as UDCA and NAM can alleviate the pathology of PD (ClinicalTrials.gov).

## Data Availability

N/A
